# Pregnancy testing in patients undergoing radiation therapy

**DOI:** 10.3332/ecancer.2017.753

**Published:** 2017-07-19

**Authors:** Shivam M Kharod, Julie Greenwalt, Camille Dessaigne, Anamaria Yeung

**Affiliations:** 1 Department of Radiation Oncology, University of Florida College of Medicine, Gainesville, Florida; 2 Department of Surgery, University of Florida College of Medicine, Gainesville, Florida

**Keywords:** radiation oncology, radiation therapy, pregnancy testing

## Abstract

Radiation therapy (RT) can be lethal to a developing fetus; therefore, determining pregnancy status before RT is essential. We here sought to determine how many women treated with RT at our institution for over one year were at risk for pregnancy when starting RT. We retrospectively reviewed the medical records of all female patients 12–55 years old treated with radiation, i.e. 1 October 2012 to 31 September 2013. Patients were categorised as ‘at risk’ if they had a uterus and ‘no risk’ if they had a hysterectomy. Documented birth control, pregnancy test status, and timing of the pregnancy test in relation to the radiation start date were recorded.

We included 131 female patients with a median age of 48 years (range 14–55 years). Breast cancer was the most prevalent disease site (18%) followed by head/neck and central nervous system (both 11%). Of the 131 patients, 35 were deemed ‘no risk’ and 95 (72%) were ‘at risk’. Pregnancy testing of the ‘at risk’ population was done in 47%, but only 17% of the pregnancy testing was performed accurately, which we defined as a test performed within 14 days before starting RT. Over one year, 66% (63/95) of ‘at risk’ women were not tested appropriately before starting RT. Most (66%) women of child-bearing age with an intact uterus receiving RT at our institution were not appropriately tested for pregnancy before the initiation of RT. These data laid the foundation for our formal pregnancy testing policies for women undergoing RT.

## Background/introduction

Despite the potentially devastating consequences of exposing a developing fetus to high doses of ionising radiation, pregnancy screening guidelines have not been established and implemented in the field of radiation oncology. Thus, the purpose of this study was to determine how many women treated with radiotherapy at our institution during one year were at risk for pregnancy before starting RT and to develop appropriate pregnancy screening guidelines.

## Methods

We retrospectively reviewed the medical records of all female patients between 12 and 55 years old at the time of treatment who were treated with external-beam RT, brachytherapy, or radioactive iodine from 1 October 2012, to 31 September 2013. During this time interval, there was no standardised pregnancy screening policy in place in our department of Radiation Oncology. Patients were categorised as ‘at risk’ if they had a uterus and ‘no risk’ if they had a hysterectomy documented in their chart. All women were considered to be premenopausal in this study as formal menopausal testing or information was not available. Pregnancy testing could consist of either blood or urine testing. However, a pregnancy test was deemed ‘appropriate’ if the test was performed fewer than 15 days before starting radiation and ‘not appropriate’ if the test was performed 15 or more days before radiation.

## Results

### Patient and treatment characteristics

Between 1 October 2012, and 31 September 2013, 131 female patients with a median age of 48 years (range 14–55 years) met the inclusion criteria. Thirty-five patients (26%) were 40 years old or younger, and 14 patients were 30 years old or younger. Breast cancer was the most common cancer type in our population (18.3%) followed by head-and-neck cancers (11.4%) and central nervous system cancers (11.4%). The median age for patients with breast cancer was 50 years (range 33.3–56 years), 52 years for head and neck cancers (range 34–55.9), and 34 years for CNS cancers (range 14.6–53.8). The youngest subgroup populations by cancer type were heterotopic bone tumours, with a median age of 29 years, followed by central nervous system cancers with a median age of 34 years, and thyroid cancers with a median age of 36 years.

The most common form of radiation delivered was external-beam RT followed by radioactive iodine and brachytherapy. Overall, 120 patients were treated with curative intent while 11 were treated palliatively.

### Risk status

Of the 131 female patients in our study, 35 patients (27%) were deemed ‘no risk’ because of a documented hysterectomy, while 95 (72%) were deemed ‘at risk’. One patient’s uterus status was unknown.

### Pregnancy screening

Of the 95 patients ‘at risk’ for pregnancy, 45 (47%) received pregnancy screening in the form of blood or urine tests, while 50 (53%) did not receive any type of pregnancy testing (Table 1). However, of the 45 patients who received pregnancy screening in the ‘at risk’ group, only 16 received screening within 14 days of the start of radiation, or what we deemed to be an ‘appropriate’ test. Thus, only 16 of the 95 ‘at risk’ patients (17%) received an ‘appropriate’ pregnancy test, meaning that 79 of the 95 ‘at risk’ patients (83%) did not receive an appropriate pregnancy test before starting RT. Of these 16 ‘at risk’ patients who received an ‘appropriate’ pregnancy test, nine had this testing done because of recent surgery.

### Birth control measures

Of the 131 patients, 16 (12%) had documented birth control measures; seven had a previous tubal ligation and three were taking oral contraceptive pills. These patients were in the ‘at risk’ category as they had a uterus at the time of treatment. None of the patients that had documented birth control received an appropriate pregnancy test. When these 16 patients with documented birth control are included in the analysis, 63 of the 95 (66%) ‘at risk’ patients were neither on birth control measures nor ‘appropriately’ tested.

For the 50 ‘at risk’ patients who did not receive pregnancy testing at any time, the patients that did not receive screening with respect to all patients of their subsite varied by the subsite for which they were being treated. Overall, 70% of ‘at risk’ patients being treated for sarcoma, 60% of ‘at risk’ patients being treated for head-and-neck cancers, 57% of ‘at risk’ patients being treated for lung cancer, and 33% of ‘at risk’ patients being treated for breast cancer and CNS cancers did not receive pregnancy testing of any sort. No patients with thyroid cancer that were ‘at risk’ went unscreened.

## Discussion

Our study found that an unacceptably high rate of women of reproductive age were undergoing radiation treatment without first ascertaining their pregnancy status. Of the 95 patients with the ability to become pregnant (our ‘at risk’ group), only 16 patients (17%) underwent an appropriate pregnancy test within two weeks of the start of radiation. The number of ‘at risk’ patients who were not on birth control and had either no pregnancy testing or testing done more than 14 days before the start of RT was 63; therefore, 66% of our ‘at risk’ patients underwent radiation without reliable contraception or pregnancy testing on file. We did not assess the reliability of birth control use; therefore, it is likely that even these patients were at considerable risk of pregnancy since many went untested.

It is well-known that exposing a developing human fetus to radiation can be devastating. Much of our knowledge on this subject comes from studies of atomic bomb survivors in Hiroshima and Nagasaki and from patients who were exposed to radiation before the risks of radiation were fully understood. In the cases of Hiroshima and Nagasaki, exposure to radiation greater than 100 mGy within 15 days of gestational age resulted in termination of the pregnancy owing to the overwhelming effect that radiation has on a single cell or small group of cells. Microcephaly was observed for up to 15 weeks after ovulation in a dose-dependent manner without an identifiable threshold dose, while mental retardation was most profound between weeks 8 and 25 for doses as low as 0.3 Gy [1–3]. Studies analysing exposure to medical radiation have found similar risks [4]. Dekaban found that irradiation causes severe abnormalities in fetal organ development between 4 and 11 weeks and growth abnormalities, mental retardation, and microcephaly between weeks 11 and 16 with similar but milder effects during weeks 16 to 25 [5]. Radiation exposure from 30 weeks onwards is unlikely to cause major abnormalities, but can result in functional impairments.

The World Health Organisation’s Selected Practice Recommendations for Contraceptive Use can be used to assess pregnancy status [6] and has been shown in a systematic review to have a negative predictive value between 99 and 100% [7]. Nevertheless, relying on this method when better alternatives are readily available is not advisable. There have been calls to establish a standardised protocol to screen for pregnancy in patients receiving radiation for both diagnostic and therapeutic purposes, and elective surgeries. In the United Kingdom in 2003, the National Institute for Health and Care Excellence released guidelines for preoperative pregnancy testing, calling for urine pregnancy screening for ‘all females of reproductive age’ [8]; however, adherence to the guidelines has not been optimal. In 2010, the National Health Service’s National Patient Safety Agency released a report indicating that 42 patients underwent a planned procedure without a documented preoperative pregnancy check, with three of these cases ending in spontaneous abortion following the procedure [9]. Since then, the National Health Service called for broader testing to include all women ages 12 to 55 years. This ‘blanket’ approach does, however, raise ethical, legal, and financial considerations as Larcher opined when joining in the call for guidelines to better screen for pregnancy preoperatively [10]. In the field of nuclear medicine, James and Warren in 2015 developed a consensus statement for Australia for pregnancy screening in patients ages 12 to 55 years undergoing nuclear medicine procedures [11]. This guideline recommended that a pregnancy test should only be obtained in patients who have reached menarche and either thought that they could be pregnant or had their last menstrual period greater than 10 days before their clinical evaluation for the intended procedure.

## Conclusions

This study shows that about two-thirds of women of reproductive age who have a uterus underwent radiation at our institution without documented, reliable contraception, or recent pregnancy testing. Because of this finding, we have developed a policy that ensures that women of reproductive age undergo a timely and accurate pregnancy test before starting radiation treatment. We now test all women between the ages of 13 and 55 years old who have not had a hysterectomy regardless of their or their partner’s birth control status. A pregnancy test must be performed within seven days of CT simulation, or if no simulation is performed, within seven days of the start of RT. Patients have the opportunity to decline pregnancy testing, and if they choose to do so, their signed declination becomes part of their medical record. By creating a protocol that screens all women of child-bearing age before starting RT, we hope to eliminate the risk of fetal irradiation.

## Conflict of interest notification

The authors declare that they have no conflict of interest.

## Figures and Tables

**Table 1.** Patient outcomes.

Outcomes	No. of “at risk” patients*
Received a pregnancy test any time prior to radiation therapy	45 (47%)
Received an appropriate† pregnancy test	16 (17%)
Had documented birth control	16 (17%)
No documented birth control AND no appropriate pregnancy test	63 (66%)

* ”At risk” is defined as women between the ages of 12 and 55 years old with a uterus intact.

† We defined an appropriate pregnancy test as being done within 14 days of the start of radiation.

## References

[1] Verreet T (2016). Current evidence for developmental, structural, and functional brain defects following prenatal radiation exposure. Neural Plast.

[2] Greskovich JF, Macklis RM (2000). Radiation therapy in pregnancy: risk calculation and risk minimization. Semin Oncol.

[3] Miller RW (1990). Effects of prenatal exposure to ionizing radiation. Health Phys.

[4] Hall E, Giaccia A (2012). Fractionated radiation and the dose-rate effect in. Radiobiology for the Radiologist.

[5] Dekaban AS (1968). Abnormalities in children exposed to x-radiation during various stages of gestation: tentative timetable of radiation injury to the human fetus. J Nucl Med.

[6] Curtis KM (2013). Adaptation of the World Health Organization's Selected Practice Recommendations for Contraceptive Use for the United States. Contraception.

[7] Tepper NK, Marchbanks PA, Curtis KM (2013). Use of a checklist to rule out pregnancy: a systematic review. Contraception.

[8] https://www.nice.org.uk/guidance/cg3/resources/preoperative-tests-for-elective-surgery-27093840325.

[9] http://www.nrls.npsa.nhs.uk/resources/?EntryId45=73838.

[10] Larcher V (2012). Developing guidance for checking pregnancy status in adolescent girls before surgical, radiological or other procedures. Arch Dis Child.

[11] James DJ, Warren-Forward HM (2015). Development of consensus statements for pregnancy screening in diagnostic nuclear medicine: a Delphi study. J Nucl Med Technol.

